# archiDART v3.0: A new data analysis pipeline allowing the topological analysis of plant root systems

**DOI:** 10.12688/f1000research.13541.1

**Published:** 2018-01-08

**Authors:** Benjamin M. Delory, Mao Li, Christopher N. Topp, Guillaume Lobet

**Affiliations:** 1 Ecosystem Functioning and Services, Institute of Ecology, Leuphana University, Lüneburg, 21335, Germany; 2 Donald Danforth Plant Science Center, St. Louis, MO, 63132, USA; 3 Agrosphäre (IBG-3), Forschungszentrum Jülich GmbH, Jülich, 52428, Germany; 4 Earth and Life Institute, Université catholique de Louvain, Louvain-la-Neuve, 1348, Belgium

**Keywords:** archiDART, plant root systems, topology, persistent homology, Fitter indices, Data Analysis of Root Tracings (DART), Root System Markup Language (RSML)

## Abstract

Quantifying plant morphology is a very challenging task that requires methods able to capture the geometry and topology of plant organs at various spatial scales. Recently, the use of persistent homology as a mathematical framework to quantify plant morphology has been successfully demonstrated for leaves, shoots, and root systems. In this paper, we present a new data analysis pipeline implemented in the R package archiDART to analyse root system architectures using persistent homology. In addition, we also show that both geometric and topological descriptors are necessary to accurately compare root systems and assess their natural complexity.

## Introduction

The quantification of plant root systems is central in many research areas, ranging from developmental studies ^1^ to crop phenotyping ^2^. Root systems are often characterised using geometric descriptors, such as the total root length ^3^, total root surface ^4^, or number of root tips ^5^. However, such descriptors often fail to describe the full complexity of root systems. Additional descriptors able to describe the topology of root systems are often missing, despite the fact that they can help understanding the feedback between plant morphology and root system functions ^6, 7^.

**Figure 1.**
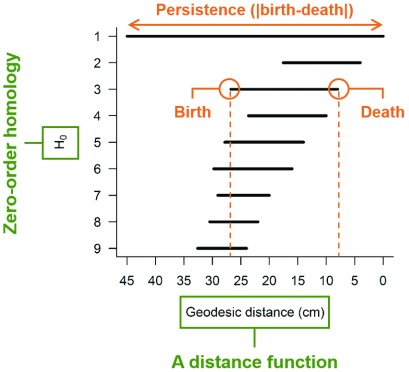
Persistence barcode capturing the topology of a plant root system. When two H _0_ branches merge, the longest one persists and the shortest one dies.

The topology of a root system is an important component of its architecture and refers to how individual roots are connected to each other through branching ^8^. Studying the topology of branching structures as complex as root systems is challenging and requires quantitative methods allowing the description and comparison of plant morphologies ^7^. In the 1980’s, A.H. Fitter introduced a method to describe branching structures and classify root systems into topologically distinct networks ^9^. His method relies on the calculation of three indices to describe the topology of a root system, namely the magnitude, altitude, and external path length. A detailed description of this method can be found in 9, 10. More recently, the use of persistent homology to quantify plant morphologies was introduced in the plant sciences community. This method was successfully used to quantify leaf shapes, leaf serrations, and root system architectures ^11^. Persistent homology is a mathematical framework allowing the quantification of plant morphologies at different scales (from organs to organisms). Because plant roots can be represented as a succession of nodes connected by straight lines in a tree graph, they are referred to as zero-order homology groups (H _0_, path-connected component) in mathematics. The goal of a persistent homology analysis applied to a root system is to study how H _0_ features persist across the scales of a continuous mathematical function. A common mathematical function used to capture the topology of branching structures, such as plant shoots and root systems, is the geodesic distance (i.e., the distance measured along the roots between the root system base and any point of the root system). A nice explanation of how persistent homology can be applied to capture plant topologies is provided in 12. The main output of a persistent homology analysis is a persistence barcode recording the birth (apparition of a new connected component) and death (fusion of two connected components) of each H _0_ branch when a distance function traverses the branching structure ( Figure 1). The degree of similarity between different root system topologies can be assessed by computing a pairwise distance matrix using a bottleneck distance method to compare persistence barcodes. Multivariate statistical tools, such as multidimensional scaling, can then be used to visualize topological differences between root systems ^12^.

In the past decade, many tools were developed to analyse root systems from digital images (for an extensive list, see the plant-image-analysis.org website ^13^) or model root system architectures ^14, 15^. Several of these tools are able to extract the full root system architecture from the images, including the topology ^16– 19^. A common format for the storage of root architecture data, the Root System Markup Language (RSML) ^20^, was also created to facilitate the exchange of information between researchers. Building on this new format, several tools were created to analyse root architecture data ^20, 21^. Among these tools, the R package archiDART offers a wide range of functionalities to analyse root system architectures in a free, open-source, and popular data analysis environment ^21^.

In this paper, we present a new version (v.3.0) of the R package archiDART. In comparison with the version described earlier ^21^, this version now includes several topological analysis methods, including, but not limited to, persistent homology. Our main objective is to demonstrate how the functions of the archiDART package can be used to analyse and compare the topology of plant root systems using persistent homology. In addition, we also aim to show that the topological analysis of plant root systems is highly complementary to the more classical approach that uses a set of geometric descriptors to compare root systems.

## Methods

### Implementation

archiDART is an R package developed for the automated analysis of plant root system architectures using Data Analysis of Root Tracings (DART) ^17^ and Root System Markup Language files (RSML) ^20^. The version 3.0 of archiDART can be downloaded from the CRAN repository. An overview of the functions available in the package is presented in Table 1. Among the 10 functions developed for the package, 5 were already presented elsewhere ^21^ and will not be further discussed in this paper.

**Table 1.** Summary of the functions available in the archiDART package version 3.0. The italicized functions were already presented in 21. DART, Data Analysis of Root Tracings; RSML, Root System Markup Language.

R functions	Description	Input data	Returned R objects	Version
*architect*	Computing traits describing the global root system architecture	DART and RSML files dartToTable and rsmlToTable objects	Data frame	1.0
*archidraw*	Plotting vectorized root systems	DART and RSML files	Plot window	1.0
*archigrow*	Computing growth rates and plotting vectorized root systems	DART and RSML files	List and plot window	1.0
*latdist*	Computing lateral root length and density distribution	DART and RSML files	List	1.0
*trajectory*	Computing root growth directions and trajectories	DART and RSML files	List and plot window	1.0
rsmlToTable	Import RSML files into a single data frame	RSML files	rsmlToTable object (data frame)	3.0
dartToTable	Import DART files into a single data frame	DART files	dartToTable object (data frame)	3.0
perhomology	Topological analysis using persistent homology	dartToTable and rsmlToTable objects	perhomology object (list). Each element of the list is a barcode object (matrix).	3.0
plot.barcode	Plot the persistence barcode (S3 method)	barcode object	Plot window	3.0
bottleneckdist	Computing a pairwise bottleneck distance matrix	perhomology object	Matrix	3.0

In comparison with the version presented earlier, the version 3.0 of the package supports the analysis of 3D root systems. In addition, time series data in RSML files can be analysed if the root system age is stored as a continuous function along the root segments. Finally, we developed a set of 5 new functions and updated the architect function to allow the topological analysis of plant root systems. The architect function is now able to calculate the topological indices introduced by Fitter ^10^, and the 5 new functions presented in this paper are devoted to the topological analysis of root systems using persistent homology ^12^.

### Operation

All functions of archiDART were coded using the R programming language. The package is compatible with Windows, Mac OS X, and major Linux operating systems. A detailed documentation file listing the package dependencies and describing all the functions listed in Table 1 can be downloaded from the CRAN package area. The bottleneckdist function of archiDART relies on the bottleneck function of the TDA package ^22^ to compute the bottleneck distance between two persistence diagrams.

### Root system library

The root system library used in this paper has already been presented elsewhere ^23^. Briefly, this library contains a total of 10,464 simulated root systems created using the root architecture model ArchiSimple ^14^. The result of each simulation was stored as an RSML file. The library consists of two categories of root systems: tap-rooted (5212) and fibrous (5252). For the use cases presented in this paper, 50 tap-rooted and 50 fibrous root systems were selected from the RSML library. All root systems used in this paper had a total root length comprised between 17 and 23 m (20 m ± 15%). Summary statistics describing the root system library used in this study are presented in Table S1.

### archiShiny: A web application demonstrating the capabilities of archiDART

In order to demonstrate and illustrate the capabilities of archiDART, we developed a web application (archiShiny) using the Shiny library ^24^. This application is freely available here: https://plantmodelling.shinyapps.io/archidart. We developed archiShiny with the following aims in mind: (1) demonstrating how multivariate statistical tools (such as principal component analysis) can be used on the aggregated metrics computed by the architect function to differentiate root systems; (2) showing how root systems can be plotted using the advanced graphical functions of the ggplot2 library ^25^; and (3) comparing the topology of root systems using persistent homology. The web application uses a library of 70 RSML files created using the root architecture model ArchiSimple ^14^. Based on the initial values of the parameters of the model, the root systems were classified into seven genotypes (mock, dense, sparse, steep, shallow, slow, and fast). Each genotype was represented by 10 simulations. The different genotypes were based on a standard parameter set (mock) and had one parameter changed: growth rate (slow vs. fast), inter-lateral distance (dense vs sparse) or gravitropism (steep vs shallow).

## Use cases

**Figure 2.**
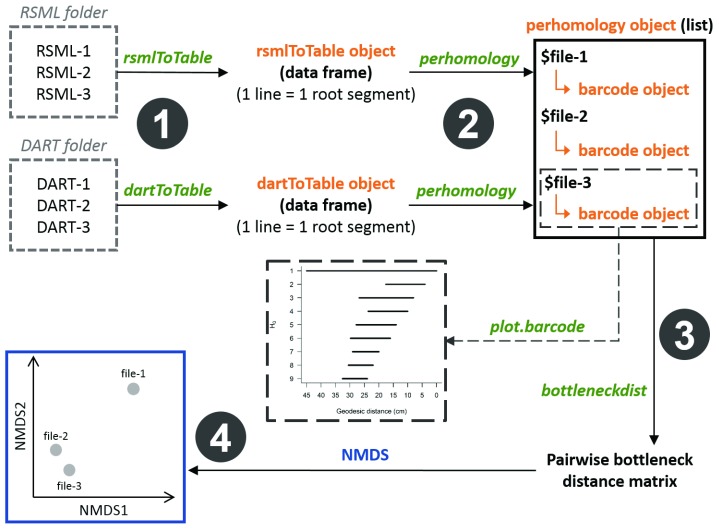
Analysis pipeline used in archiDART to compare the topology of plant root systems using persistent homology. The archiDART functions are italicized and written in green. archiDART objects are written in orange. DART, Data Analysis of Root Tracings; RSML, Root System Markup Language; NMDS, non-metric multidimensional scaling.

After package installation, the topological analysis of plant root systems (RSML files) using persistent homology comprises four main steps: (1) creating an rsmlToTable object, (2) computing persistence barcodes, (3) computing a pairwise bottleneck distance matrix, and (4) visualizing topological differences between root systems using non-metric multidimensional scaling (NMDS). The main steps of the analysis performed in this section of the paper are summarized in Figure 2. Although we only present the analysis pipeline developed for RSML files, root systems vectorized with DART can be analysed using exactly the same approach (see Figure 2).

### Creating an rsmlToTable object

The first step of the analysis is to import the RSML files into R with the rsmlToTable function. If root systems were vectorized with DART, the dartToTable function should be used instead. The rsmlToTable function creates a data frame (table) containing at least 23 columns (spatial coordinates, length, diameter, surface, volume, growth rate, orientation, geodesic distance, etc.) and as many lines as root segments. Here, a root segment is defined as the straight line between two nodes in the data file. The table is an rsmlToTable object that can directly be used as an input to compute the persistence barcodes using the perhomology function. It is worth noting that rsmlToTable objects can also be used as an input for the architect function of this new version of the package to compute a set of aggregated metrics describing the global architecture of plant root systems.

### Computing the persistence barcodes

**Figure 3.**
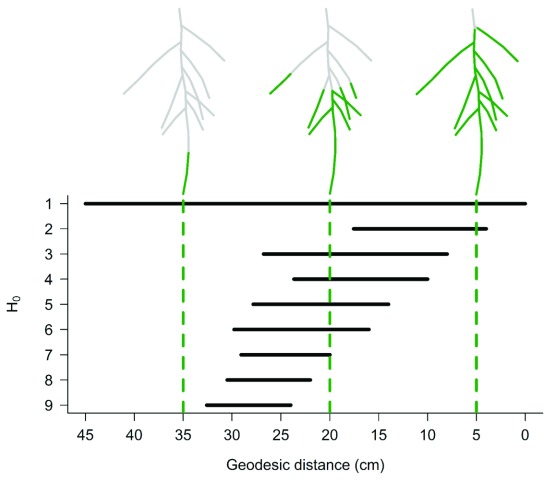
Persistence barcode of the topology of a plant root system computed using a geodesic distance function. Vertical lines indicate the position along the geodesic distance function (from left to right). The version of this figure in the online article is interactive and was produced with the plotly library ^26^.



                        
The perhomology function computes the persistence barcode of each root system stored in an rsmlToTable or a dartToTable object. Each persistence barcode is computed using a geodesic distance function ( Figure 3). For each root system, the results are stored as a barcode object in a list that contains as many elements as root systems. A barcode object is a matrix with 3 columns (dimension, birth, and death) and has as many lines as zero-order homology bars in the persistence barcode. An S3 method (plot.barcode) was developed for plotting persistence barcodes. A code example to compute and plot persistence barcodes from RSML files is provided below.

                        path <- "PATH_TO_FOLDER_WITH_RSML_FILES"
table <- rsmlToTable(path, fitter=TRUE)
ph <- perhomology(table)
plot(ph$RSML_NAME)
                    


### Computing a pairwise bottleneck distance matrix

To compare persistence barcodes against each other, a pairwise distance matrix is needed and the bottleneck distance is one possible option. The bottleneck distance is considered as a robust dissimilarity metric between two persistence barcodes, and its interpretation is quite straightforward: the greater the distance between two persistence barcodes, the greater will be the dissimilarity between them ^12^. Such pairwise bottleneck distance matrix can be calculated with the bottleneckdist function of the package. This function only requires a perhomology object as an input. It has to be noted that the computation time required to compute a bottleneck distance matrix is highly dependent on the number and complexity of root systems being compared. A code example to compute a bottleneck distance matrix from persistence barcodes is provided below.



                        
                        path <- "PATH_TO_FOLDER_WITH_RSML_FILES"
table <- rsmlToTable(path, fitter=TRUE)
ph <- perhomology(table)
dist <- bottleneckdist(ph)
                    


### Persistent homology: An efficient method allowing the topological analysis of plant root systems

A large variety of morphological, architectural, and topological traits can be measured on plant root systems (e.g., total root length, diameter, number of lateral roots per branching order, lateral root density, Fitter indices, etc.). When working with root architecture models and image analysis tools supporting the RSML format, such traits can be easily extracted from RSML files using the architect function of the archiDART package ^21^ or the ImageJ plugin RSML Reader ^20^. Using multivariate statistical tools, such as principal component analysis (PCA), one can then determine the key traits differentiating the root systems being compared ^23^. In the next section, we would like to show that the information gained with this approach can be nicely complemented by a topological analysis of root systems using persistent homology.

**Figure 4.**
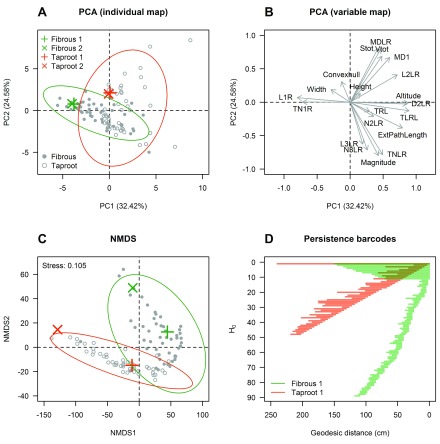
Two complementary approaches for comparing root system architectures. In total, 100 root systems were considered for the analysis (50 fibrous and 50 tap-rooted). In the first approach, root systems were compared using a set of 20 traits computed by the architect function of archiDART. A PCA was then performed to visualize differences between root systems and find the most interesting traits to differentiate them (panels **A** and **B**). The PCA was performed on a correlation matrix constructed from scaled variables using the PCA function of the FactoMineR package ^28^. In the second approach, we used persistent homology to compare the topology of root systems. Topological differences between root systems were visualized using non-metric multidimensional scaling (NMDS, panel **C**). The NMDS was performed on a pairwise bottleneck distance matrix with the metaMDS function of the vegan library ^29^. In panel **D**, two persistence barcodes are compared. In panels **A** and **C**, each dot is a branching structure and four root system of interests are spotted using orange (taproot) and green (fibrous) crosses. Abbreviations used in panel **B**: TRL, total root length; L1R, total first-order root length; TN1R, number of first-order roots; TNLR, total number of lateral roots; TLRL, total lateral root length; N2LR, number of second-order roots; N3LR, number of third-order roots; L2LR, total second-order root length; L3LR, total third-order root length; MD1, mean first-order root diameter; MDLR, mean lateral root diameter; D2LR, second-order root density; Convexhull, convex hull area; Stot, total root surface area; Vtot, root system volume; ExtPathLength, external path length.

After selecting 100 root systems from a large RSML library, we first used the architect function to compute a set of 20 traits for each root system ( Table S1). Then, we performed a PCA to visualize differences between root systems and find the most interesting morphological, architectural, and topological variables to differentiate them. On the score plot constructed with the two first principal components, a good separation between fibrous and taproot root systems can be observed on the first principal component ( Figure 4A). On average, fibrous root systems were characterized by a greater number/length of first-order roots, while taproot systems had a greater lateral root length and a greater secondary root density ( Figure 4B, Table S2). Interestingly, two topological indices (altitude and external path length) were on average greater for taproot systems. On the second principal component, however, a separation between dicotyledonous root systems can be observed ( Figure 4A). Negative PC2 scores were mainly associated with taproot systems having a greater number/length of tertiary roots and a greater magnitude, while root systems with positive PC2 scores had on average greater root diameters, surface, and volume ( Figure 4B, Table S2). Although this approach is very useful to assess root system diversity and derive a functional classification of root systems ^27^, it poorly takes into account topological differences that might exist between root systems sharing similar trait values.

**Figure 5.**
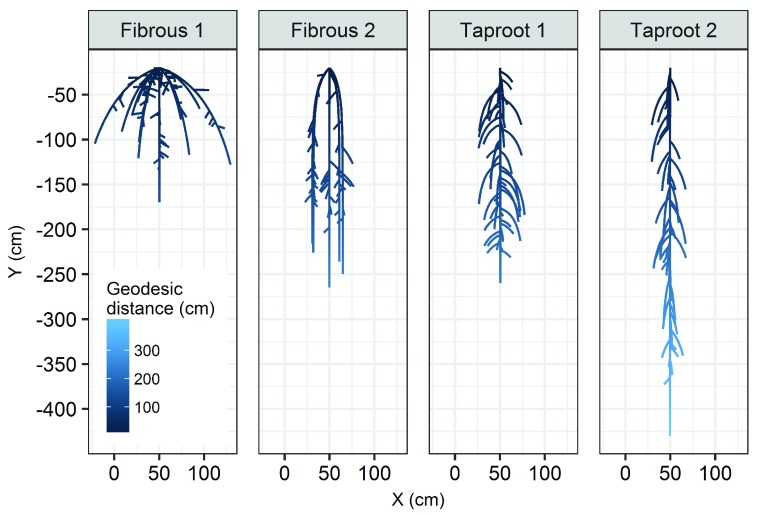
Representative root systems highlighted in Figure 4 (colour crosses). The colour code refers to the geodesic distance (cm).

To illustrate this, we plotted four representative root systems from the RSML library used in this study ( Figure 5). Although the global architecture and topology of these root systems clearly differ, fibrous 1 and fibrous 2, as well as taproot 1 and taproot 2, were poorly separated by the PCA ( Figure 4A). Therefore, we performed a topological analysis of the root systems in our library using the persistent homology analysis pipeline described previously. Non-metric multidimensional scaling (NMDS) was used to visualize dissimilarities between persistence barcodes ( Figure 4C and D). Results showed that (1) fibrous and taproot root systems can be clearly separated using persistent homology, and (2) strong topological differences exist between fibrous 1 and fibrous 2, as well as between taproot 1 and taproot 2, despite the fact that these root systems were not separated by the PCA. Altogether, these results showed that persistent homology is highly complementary to the more traditional approach consisting at using a set of aggregated metrics to compare root systems.

## Conclusions

In this paper, we presented a new analysis pipeline implemented in the R package archiDART to perform topological analysis of plant root systems using root architectural data (DART or RSML files). Using root architecture models, we showed that persistent homology is an efficient tool to capture and compare the topology of a large diversity of root systems. In addition, our results showed that the use of both geometric and topological descriptors are necessary to capture the natural complexity of plant root systems. Because topology is independent of transformation and deformation, the analysis pipeline described in this paper is highly flexible and can be used on data describing the architecture of 3D (e.g., root architecture models) and 2D (e.g., excavated root systems) root systems. Altogether, we believe that this great flexibility in root architecture data, the ease of use of the functions developed for the analysis pipeline presented in this paper, as well as the open-source nature of archiDART, make topological analysis of root systems widely accessible to the scientific community.

## Data and software availability

The data and R codes used for the use cases presented in this manuscript are available: https://doi.org/10.5281/zenodo.1117836 ^30^

### archiDART

The latest stable version of archiDART is available on the CRAN repository: http://cran.r-project.org/package=archiDART

Source code available from: https://github.com/archidart/archidart

Archived source code as at time of publication: https://dx.doi.org/10.5281/zenodo.1117864 ^31^

Software license: GNU GPL v2.0

More information about archiDART can be found on this website: https://archidart.github.io/

### Web application

The web application illustrating the capabilities of archiDART is accessible at: https://plantmodelling.shinyapps.io/archidart

The data and codes used to make the web application are available: https://github.com/archidart/archishiny

Archived source code as at time of publication: https://doi.org/10.5281/zenodo.1133435 ^32^

License for web application: GNU GPL v3.0

## Supplementary material

**Table S1. Descriptive statistics of the root system library used in this study.** The RSML library consisted of 50 fibrous and 50 taproot root systems created using the ArchiSimple model ^14^.

**Table S2. Principal component analysis: Correlation between each root system variable and the two first principal components.** Correlation coefficients written in bold contributed significantly to a principal component (PC). We considered that a variable contributed significantly to a PC if its contribution (in %) was greater than the contribution that would have been expected if all variables contributed equally to a PC (threshold value equal to 5%). The topological indices used in the PCA were calculated following ^9, 10^.
