# Supplementary material for: archiDART v3.0: A new data analysis pipeline allowing the topological analysis of plant root systems
Source: F1000Res. 2018 Jan 8;7:22. [Version 1] doi: 10.12688/f1000research.13541.1 (PMC5871803; doi:10.12688/f1000research.13541.1)
Supplement: Supplementary file 1 [file f1000research-7-14706-s0000.tgz › 79c16ddf-1b57-47d6-acde-24ef6ccde867.docx]

**Table S1. Descriptive statistics of the root system library used in this study.** The RSML library consisted of 50 fibrous and 50 taproot root systems created using the ArchiSimple model (14).

| **Variable** | **Fibrous** | | | | **Taproot** | | | | **Unit** |
| --- | --- | --- | --- | --- | --- | --- | --- | --- | --- |
|  | **Min** | **Max** | **Mean** | **SD** | **Min** | **Max** | **Mean** | **SD** |  |
| Total root length | 1716.4 | 2283.8 | 2006.0 | 179.5 | 1715.8 | 2290.1 | 1992.6 | 199.3 | cm |
| Total first-order root length | 105.0 | 1835.0 | 780.9 | 424.0 | 135.0 | 410.0 | 263.3 | 66.3 | cm |
| Number of first-order roots | 1 | 18 | 6.4 | 3.8 | 1 | 1 | 1 | 0 | **-** |
| Total number of lateral roots | 33 | 218 | 90.5 | 37.0 | 40 | 205 | 92.6 | 38.8 | **-** |
| Total lateral root length | 364.1 | 1994.1 | 1225.1 | 423.7 | 1325.7 | 2140.0 | 1729.3 | 218.6 | cm |
| Number of second-order roots | 33 | 174 | 76.5 | 29.3 | 26 | 140 | 57.1 | 23.5 | - |
| Number of third-order roots | 0 | 84 | 14.0 | 23.1 | 0 | 115 | 35.5 | 37.9 | - |
| Total second-order root length | 364.1 | 1994.1 | 1112.6 | 388.3 | 565.0 | 2140.0 | 1339.8 | 439.8 | cm |
| Total third-order root length | 0 | 621.6 | 112.4 | 192.1 | 0 | 1314.7 | 389.5 | 441.3 | cm |
| Mean first-order root diameter | 2.04 | 3.98 | 2.86 | 0.56 | 3.35 | 45.16 | 9.13 | 7.70 | mm |
| Mean lateral root diameter | 0.75 | 2.58 | 1.37 | 0.42 | 0.91 | 8.26 | 2.16 | 1.49 | mm |
| Second-order root density | 0.02 | 0.71 | 0.16 | 0.15 | 0.11 | 0.65 | 0.23 | 0.12 | roots/cm |
| Height | 100.0 | 360.0 | 176.9 | 52.5 | 135.0 | 410.0 | 263.3 | 66.3 | cm |
| Width | 26.3 | 189.3 | 65.6 | 33.4 | 24.0 | 133.6 | 58.8 | 20.9 | cm |
| Convex hull area | 1882.3 | 24745.3 | 7124.8 | 3943.9 | 4025.9 | 22806.1 | 9469.2 | 3539.3 | cm² |
| Total root surface area | 790.2 | 1894.1 | 1245.3 | 310.5 | 767.3 | 6593.9 | 1841.1 | 1197.8 | cm² |
| Root system volume | 27.8 | 152.7 | 72.9 | 33.5 | 35.2 | 4356.5 | 384.0 | 750.3 | cm³ |
| Magnitude | 45 | 220 | 96.9 | 35.8 | 41 | 206 | 93.6 | 38.8 | - |
| Altitude | 6 | 97 | 28.9 | 20.8 | 27 | 141 | 58.1 | 23.5 | - |
| External path length | 149 | 6699 | 1417.9 | 1373.3 | 901 | 12870 | 2805.5 | 2133.0 | - |
