# Supplementary material for: archiDART v3.0: A new data analysis pipeline allowing the topological analysis of plant root systems
Source: F1000Res. 2018 Jan 8;7:22. [Version 1] doi: 10.12688/f1000research.13541.1 (PMC5871803; doi:10.12688/f1000research.13541.1)
Supplement: Supplementary file 2 [file f1000research-7-14706-s0001.tgz › 3c188252-0165-49d8-91e8-509d03539aeb.docx]

**Table S2. Principal component analysis: Correlation between each root system variable and the two first principal components.** Correlation coefficients written in bold contributed significantly to a principal component (PC). We considered that a variable contributed significantly to a PC if its contribution (in %) was greater than the contribution that would have been expected if all variables contributed equally to a PC (threshold value equal to 5%). The topological indices used in the PCA were calculated following (9,10).

| **Variable** | **PC1** | **PC2** |
| --- | --- | --- |
| Total root length | 0.30 | -0.14 |
| Total first-order root length | **-0.80** | 0.07 |
| Number of first-order roots | **-0.72** | 0.00 |
| Total number of lateral roots | 0.49 | **-0.79** |
| Total lateral root length | **0.89** | -0.13 |
| Number of second-order roots | 0.36 | -0.21 |
| Number of third-order roots | 0.26 | **-0.72** |
| Total second-order root length | **0.71** | 0.41 |
| Total third-order root length | 0.19 | **-0.63** |
| Mean first-order root diameter | **0.60** | **0.67** |
| Mean lateral root diameter | 0.47 | **0.80** |
| Second-order root density | **0.86** | -0.03 |
| Height | 0.16 | 0.13 |
| Width | -0.29 | 0.19 |
| Convex hull area | -0.13 | 0.31 |
| Total root surface area | 0.42 | **0.80** |
| Root system volume | 0.48 | **0.69** |
| Magnitude | 0.43 | **-0.80** |
| Altitude | **0.89** | -0.01 |
| External path length | **0.79** | -0.39 |
