# Supplementary material for: archiDART v3.0: A new data analysis pipeline allowing the topological analysis of plant root systems
Source: F1000Res. 2018 Jan 8;7:22. [Version 1] doi: 10.12688/f1000research.13541.1 (PMC5871803; doi:10.12688/f1000research.13541.1)
Supplement: Peer review history

# Peer review: Referee response for version 1

**DOI:** 10.5256/f1000research.14706.r30873

**Published:** 2018-03-26

**Authors:** Nathan D. Miller

**Affiliations:** 1 Department of Botany, University of Wisconsin-Madison, Madison, WI, USA

"archiDART v3.0: A new data analysis pipeline allowing the topological analysis of plant root systems”, presents new analysis methods available in with the archiDART platform.

**Constructive Criticisms:**

 As is often the case with multivariate analysis, we reach for our favorite or most comfortable analysis from the myriad of linear alphabet soup. In this case, the authors choose principal component analysis (PCA) to “… determine the key traits differentiating the root systems being compared“. When using linear algebra tools for finding separation of groups based on multiple traits, the authors should consider using a discriminant style analysis such as Linear Discriminant Analysis (LDA), Multi-class LDA (mLDA), or Partial Least Squares Discriminant Analysis (PLS-DA). Unlike PCA, these methods include group structure in the objective function resulting in an answer which is closer to the authors goal.

 The authors need to take care of the scale or units of the traits when using PCA. For example, if some traits yield numbers on the order of 10^3 and others yield numbers on the order of 10^-3, how would the authors expect this to affect the presented analysis? Similar care needs to be taken when using LDA/mLDA, perhaps a normalization such as z-score or min-max would overcome the problem. As the objective function for PLS-DA has z-score “built” in, it might most easily yield reasonable results.  

 The authors chose to use non-metric multidimensional scaling (NMDS), on the pairwise distance matrix for the persistent homology analysis and PCA for the trait-style analysis. If there a way to present a common approach between these two methodologies?  

**Complements:**

 Simply and most importantly, the authors meet their goal. They present a free, open source, and usable R package for persistent homology analysis and useful accompanying graphical abilities. 

**Conclusions:**

 While a more thorough compare/contrast workup could be presented between the multivariate and persistent homology approaches, this paper does not claim to handle the task. The authors should consider softening the expectations/phrasing of PCA as a discrimination method or use a different approach.

I have read this submission. I believe that I have an appropriate level of expertise to confirm that it is of an acceptable scientific standard.

# Peer review: Referee response for version 1

**DOI:** 10.5256/f1000research.14706.r29567

**Published:** 2018-03-12

**Authors:** Alexander Bucksch

**Affiliations:** 1 Department of Plant Biology, Warnell School of Forestry and Natural Resources, Institute of Bioinformatics, University of Georgia, Athens, GA, USA

Delory et al. present an extension package for ArchiDart that allows the user to compute topological descriptors of branching plant shapes. The contribution is valuable for the community of researchers studying plant morphology. I do recognize that this was a lot of work to implement.

 I have several suggestions to improve the paper for the readers and the user community.

1. The paper gives overall a good intuition of how topological indices of branching structures are computed. Yet, the detail for further exploration is missing. Looking at the code and the paper, it was unclear to me how a user can change the function used to expand over the surface of the branching structure. Currently only the geodesic distance is used to extract a homology of a given branching structure. Does the code provide an interface to choose the function? In my opinion, It would be good to have a pseudo code to the paper that allows to see into the many functions used in the R implementation.
2. The capabilities of the software were demonstrated nicely on simulated branching structures. While the results are convincing for “perfect data” I missed a validation on noisy and incomplete data. In other words, I couldn’t find evidence on the robustness of the method and if it would translate to branching structures extracted from imaging data. It would be good to know for the reader which quality criteria apply to the input data and what are the limitations if data is partial. That way the code becomes useful for many more applications.
3. I do appreciate the contribution a lot and know the previous papers referenced in the manuscript. However, for a new reader it is very difficult to follow all the cross-references in the paper. I would like to see a one or two sentence summary of concepts for each referenced paper that contributes to the content of the presented software paper. For example: “A nice explanation of how persistent homology can be applied to capture plant topologies is provided in ^12^” -> Until here it is not clear what persistent homology is, nor the concept of homology groups is explained sufficiently. The reader needs to read to more papers in order to understand H _0_ groups etc. In part this can be resolved by being clear in the preceding description of a graph and how the geodesic distance is measured on it. It is left to the reader to assume an unknown embedding of the graph in to a metric space. Currently, the graph is only introduced by having nodes and edges. Intuitively I would expect the distance between two nodes to be the number of edges between to nodes. Yet later on it is talked about the geodesic distance on the graph. I assume that the graph is embedded in an Euclidean space and the “straight edges” might be curves for which the curve length is taken to calculate the geodesic distance between any two nodes. Or did I got this wrong? More precision in the mathematical language would be helpful. Similar issues are present elsewhere in the manuscript whenever the explanation is referenced to another paper, e.g. to Archidart 1.0.
4. A confusing aspect of the paper is that Archidart 1.0 has Archidart 3.0 as a follow up. Did I miss something? You state that Archidart 3.0 implements 5 new functions compared to Archidart 1.0. None of the functions presented in table 1 refer to Archidart 2.0. Should the presented version be Archidart 2.0?
5. A very general comment: It is nothing new that geometry and topology are needed to quantify shapes. Work of Biassotti, Spagnuolo, Veltkamp etc. show that since many years. May be most closely related to the paper here is the paper Biasotti, Silvia et al. (2008) ^1^ that discusses the interplay of geometry and topology for skeletal shape analysis. Many other papers show the same.

I have read this submission. I believe that I have an appropriate level of expertise to confirm that it is of an acceptable scientific standard, however I have significant reservations, as outlined above.

## References

[1] BiasottiSAttaliDBoissonnatJEdelsbrunnerHElberGMortaraMdi BajaGSpagnuoloMTanaseMVeltkampR: Skeletal Structures.2008; 10.1007/978-3-540-33265-7_5 145-183 10.1007/978-3-540-33265-7_5

# Peer review: Referee response for version 1

**DOI:** 10.5256/f1000research.14706.r29709

**Published:** 2018-01-26

**Authors:** Magdalena M. Julkowska

**Affiliations:** 1 King Abdullah University of Science and Technology, Thuwal, Saudi Arabia

The paper by Delory et al., "archiDART v3.0: A new data analysis pipeline allowing the topological analysis of plant root systems" describes an R package archiDART that includes methods allowing analysis root topology using persistent homology. The paper describes the novelty of the package really well and it is providing new and exciting ways for the community to differentiate between various root architecture types. The Shiny app developed is illustrating the possibilities of the new package very neatly.

 I do have few questions/suggestions:

1. When computing a pairwise bottleneck distance matrix between individual files, what kind of statistical analysis can be used to decide whether a distance between two genotypes/groups is statistically significant? The authors could at least put a suggestion of the follow-up analysis. That would be extremely useful for the part of a scientific community that works with mutants/introgression lines and examines root architecture to answer hypothesis driven questions.
2. The authors compare the classical root traits with persistent homology using PCA and non-metric multidimensional scaling and showing that the later is resulting in clear differences between fibrous and tap-root root types. I think that this difference could result in using PCA vs NMDS rather than the input. For the fair comparison, the value of two different inputs in distinguishing between two different root types, should be compared using the same method for reduction in dimensionality. 
3. Although the persistent homology is an alternative way of differentiating between the root types, the method is still having limited capability in distinguishing between "mock", "shallow" and "steep" root types (as shown in the archiDart shiny app). The same types are also not being differentiated using PCA for the "classical" root phenotypes. It would be very helpful if the authors state the limitations of using the homology (like - problems with implementing the root angle) and how exactly is the persistent homology complementary to the more classical analysis of root system architecture. 
4. There is a glitch in the app when user removes the genotypes to be plotted in the tabs "archiDRAW" and archiHOMOLOGY".

I have read this submission. I believe that I have an appropriate level of expertise to confirm that it is of an acceptable scientific standard.
